# Editorial Note: Measurement and driving factors of carbon productivity in China’s provinces: From the perspective of embodied carbon emissions

**DOI:** 10.1371/journal.pone.0338756

**Published:** 2025-12-15

**Authors:** The PLOS One Editors

The *PLOS One* Editors issue this Editorial Note to inform readers that we have concerns about aspects of the article’s peer review. Readers are advised to interpret the article [1] with caution.

We regret that this was not addressed prior to the article’s publication.

To ensure the article complies with the PLOS Data Availability policy, the authors provided the underlying data in S1 File and the following information regarding the datasets:

- The input-output data are sourced from the “China Multi-Regional Input-Output Tables” published in 2002, 2007, 2012, and 2017, with the data available at: “China Multi-Regional Input-Output Tables” published by China Statistics Press Co., Ltd; and the China Statistical Information Network [2–5].
- The regional energy consumption data for 2002, 2007, 2012, and 2017, are sourced from the “China Energy Statistical Yearbook” and the data can be obtained from the China Statistical Information Network [6–9].
- Based on the regional energy balance sheet, the direct carbon emissions were calculated following the method described in [10], Equation 2.1 of Section 2.3.1.1.

## Supporting information

S1 File Source data. (ZIP)
